# BRINGING INTERACTIVITY TO FULLY ASYNCHRONOUS PROGRAMS

**DOI:** 10.1093/geroni/igac059.1703

**Published:** 2022-12-20

**Authors:** Carolyn Clevenger

**Affiliations:** Emory University, Atlanta, Georgia, United States

## Abstract

Asynchronous online learning brings both new opportunities as well as challenges to an asynchronous psychoeducation program like Tele-Savvy@Home. While asynchronous learning offers engagement flexibility for time-strapped family caregivers, a lack of scheduled meetings represents a lack of contact and interactivity, threatens accountability, and produces a high incompletion rate. To engage participants in the next generation of this asynchronous, self-paced course, we have implemented a small cohort structure (10 participants each) and created a combination of automated prompts and optional touchpoints. The cohort structure and touchpoints mimic important shared and active learning components of the facilitated sessions. Prompts include weekly messages that nudge learners with reminders of their ideal progress point and offer value propositions for staying on-time. A pacing guide allows caregivers to build the course into their schedule in advance of the course’s start. Optional touchpoints include unit-by-unit post-tests, a cohort-based discussion board, and free text reflective exercises.

